# Optimising infliximab induction dosing to achieve clinical remission in Chinese patients with Crohn’s disease

**DOI:** 10.3389/fphar.2024.1430120

**Published:** 2024-08-27

**Authors:** Kouzhu Zhu, Xiaoliang Ding, Ling Xue, Linsheng Liu, Yan Wang, Yun Li, Qinhua Xi, Xueqin Pang, Weichang Chen, Liyan Miao

**Affiliations:** ^1^ Department of Pharmacy, The First Affiliated Hospital of Soochow University, Suzhou, China; ^2^ Department of Pharmacy, Affiliated Children’s Hospital to Jiangnan University (Wuxi Children’s Hospital), Wuxi, China; ^3^ Institute for Interdisciplinary Drug Research and Translational Sciences, Soochow University, Suzhou, China; ^4^ Department of Gastroenterology, The First Affiliated Hospital of Soochow University, Suzhou, China; ^5^ National Clinical Research Center for Hematologic Diseases, The First Affiliated Hospital of Soochow University, Suzhou, China

**Keywords:** infliximab, antibodies to infliximab, Crohn’s disease, clinical remission, population pharmacokinetic model

## Abstract

**Aims:**

A strategy based on therapeutic drug monitoring and population pharmacokinetic (popPK) models would likely increase the rate of clinical remission (CR) after infliximab (IFX) induction in patients with Crohn’s disease (CD). This study aimed to evaluate the relationship between early IFX levels and antibodies to infliximab (ATI) and CR at week 14 and simulate the probability of attaining the identified exposure target.

**Methods:**

Patients with CD (n = 140) treated with IFX were enrolled to develop the popPK model. Of these, 43 moderate-to-severe patients with CD were followed up at week 14. Simulations were performed on patients with different dosage regimens and covariates.

**Results:**

IFX levels >20.08 μg/mL at week 2, >18.44 μg/mL at week 6, and >3.08 μg/mL at week 14 were linked to CR. A one-compartment model fit the data best. The covariates influencing clearance were fat free mass, albumin and ATI levels. To achieve IFX levels >20.08 μg/mL at week 2, ≥400 mg IFX was predicted to be required in over 50% patients with 45–70 kg and 35–45 g/L albumin, except for patients with 70 kg and 30 g/L albumin.

**Conclusion:**

IFX levels >20.08 μg/mL at week 2 and absence of ATI at week 14 are associated with CR. Optimising IFX induction dosing will be critical to achieve the target of early IFX levels associated with CR.

## Introduction

Infliximab (IFX) is the first tumour necrosis factor-α (TNF-α) inhibitor for treating moderate-to-severe Crohn’s disease (CD), and clinical remission (CR) is an medium-term treatment target. However, over 30% of patients do not achieve CR after the induction period (Ben-Horin and Chowers, 2014). Moreover, patients without CR are less likely to respond to a second biologic agent (Gisbert et al., 2015; Singh et al., 2018). Some pharmacokinetic (PK) issues contribute to the lack of remission after IFX induction therapy.

Many recommendations regarding IFX target concentrations come from expert consensus and guidelines (Feuerstein et al., 2017; Mitrev et al., 2017; Cheifetz et al., 2021), mostly for the maintenance phase rather than the induction phase. The widely adopted maintenance therapeutic IFX level is 3–7 μg/mL in China (Inflammatory Bowel Disease Group, Chinese Society of Gastroenterology, Chinese Medical Association, 2018). Regarding the induction period, cohort studies and post-hoc analyses of randomised controlled trials have provided limited data on the predictive value of early trough levels of infliximab (TLI) for response and/or remission in patients with inflammatory bowel disease (IBD) (Davidov et al., 2017; Kobayashi et al., 2016; Bar-Yoseph et al., 2018; Ungar et al., 2018; Vande Casteele et al., 2019; Buhl et al., 2020; Courbette et al., 2020; Dreesen et al., 2020). No association between IFX concentrations during induction in responders and primary non-responders was differed from those of previous studies on patients with CD (Billiet et al., 2017; Buhl et al., 2020). Whether a relationship between early TLI and CR requires further verification, particularly in Chinese patients with CD.

The variability in the PK of IFX is widely observed among patients (Dreesen et al., 2018). This variability can be partly attributed to factors such as weight, albumin level, and antibodies to infliximab (ATI) titres (Dreesen et al., 2018; Matsuoka et al., 2020). Targeting IFX for optimal exposure increases the rates of remission. Therefore, model-based tools have been used to optimize IFX therapy in patients with ulcerate colitis (UC) and children with IBD (Dreesen et al., 2019; Kantasiripitak et al., 2023). Unfortunately, no regimens have been recommended for induction treatment in patients with CD, especially in adults.

This prospective cohort study aimed to demonstrate the role of therapeutic drug monitoring during IFX induction in Chinese patients with active CD. Subsequently, to maximize the success of CR during induction, a population pharmacokinetic (popPK) model was developed, and simulate different dosage regimens.

## Materials and methods

### Patient population

We conducted this prospective cohort study at the First Affiliated Hospital of Soochow University between October 2020 and December 2022. Patients with CD treated with IFX were enrolled for popPK modelling. Among these, patients with CD Activity Index (CDAI) ≥220 points at baseline initiating IFX therapy were enrolled to evaluate the relationship between early IFX levels and ATI and CR at week 14. All patients received IFX induction at weeks 0, 2, and 6, followed by maintenance therapy every 8 weeks. If the administration of IFX were not done at weeks 2, 6, and 14 before the fourth infusion, infusion intervals were considered inappropriate. CR was defined as a CDAI of less than 150 points at week 14. Within the 24 h during each visit prior to IFX infusion, a total of 2 mL of peripheral whole blood were collected for measuring TLI and ATI. This study was approved by the Institutional Review Board of the First Affiliated Hospital of Soochow University. All the participants provided written informed consent.

### IFX concentration and ATI measurement

The plasma samples were divided into two portions and stored at −80°C. IFX concentration were measured after a maximum of 14 days in storage using an in-house developed and validated indirect enzyme-linked immunosorbent assay (ELISA). Briefly, TNF-α (Peprotech, Cranbury, NJ, United States) and horseradish peroxidase-labelled anti-IFX monoclonal antibodies (Bio-Rad, Kidlington, Oxford, United Kingdom) were used as the capturing antigen and detecting antibody, respectively. The quantitation range for measuring IFX levels was 0.50–40.00 μg/mL. ATI were measured after a maximum of 4 weeks in storage. A multi-tiered testing approach based on 51 patients with IFX-naïve CD was developed and validated to measure the ATI. The screening cut point was set at 1.16 (S/N). The confirmatory cut point was determined to be a decrease in the signal of more than 17.21%. The titre cut point was 1.34. Drug tolerance at the 100 ng/mL positive control was ≤12.50 μg/mL IFX. The relative sensitivity of the assay was 50 ng/mL.

### Statistical analysis

Medians and interquartile ranges (IQRs) were used to express continuous variables, while numbers and percentages were used to express categorical variables. TLI at weeks 2, 6, and 14 were compared between the CR and non-CR groups using the Mann–Whitney test. To determine the cut-off values for the TLI, receiver operating characteristic (ROC) analysis was performed using CR as a classification variable. Sensitivity, specificity, positive predictive value (PPV), and negative predictive value (NPV) were determined for CR. The independent variables associated with CR were sex, serum albumin level at baseline, and TLI and ATI at weeks 2, 6, and 14. The Logistic regression model was used for multi-variable analysis by incorporating significant univariable factors (*P* < 0.1) using the SPSS Statistics 26.0 (IBM Corp., Armonk, NY, United States). All statistical calculations were performed using GraphPad Prism^®^ version 9.0 (GRAPHPAD, San Diego, CA, United States) and IBM SPSS Statistics for Mac, version 26.0 (IBM Corp.,).

### popPK modelling

IFX concentrations and ATI data were analysed by nonlinear mixed-effects modelling using the NONMEM 7.3 software (ICON Development Solutions Inc., Dublin, Ireland). The first-order conditional estimation with the interaction method was implemented, and the M5 method was used to incorporate observations below the limit of the assay into the likelihood (Beal, 2001). The between-subject variability was assessed using an exponential model. The residual variability was described using a combined proportional and additive model. Sex, age, height and body weight at baseline were considered potential covariates. Time-varying weight, albumin, ATI, and erythrocyte sedimentation rate (ESR) were considered potential covariates. Fat free mass (FFM) incorporated sex, height and bodyweight (Janmahasatian et al., 2005). Continuous covariates were assessed using Equation 1. Categorical covariates were assessed using Equation 2. The model was constructed by individually introducing all covariates to identify those that were statistically significant for IFX PK parameters. The significance level was set at 0.05 [df = 1, change in objective function value (OFV) = 3.84]. Additionally, a stepwise procedure was employed to analyse potential covariates based on changes in the OFV. For forward selection, the significance level was set at 0.01 (df = 1, and change in OFV = 6.64), whereas for backward elimination, the significance level was set at 0.001 (df = 1, change in OFV = 10.83). The accuracy and robustness of the final model were evaluated using bootstrap methods, goodness-of-fit plots, and visual predictive checking (VPC).

$$\theta =\theta_{1}\times {\left(\frac{C\text{ovariate}}{\text{median}}\right)}^{\theta_{2}}$$ (1)

$$\theta =\theta_{1}\times \left(1+\theta_{2}\times \text{Covariate}\right)$$ (2)

### Simulation

The developed popPK model was used to perform the simulations. One thousand patients were simulated for each tested dose regimen, and summary statistics of the probability of attaining the identified exposure targets were generated. Patient factors were set (absence of ATI, weight 45–70 kg, and albumin 30–45 g/L), and only the factors being explored were allowed to vary. Simulation patients reflected most patients characteristics seen in clinical practice.

## Results

### Patient characteristics

**FIGURE 1:**
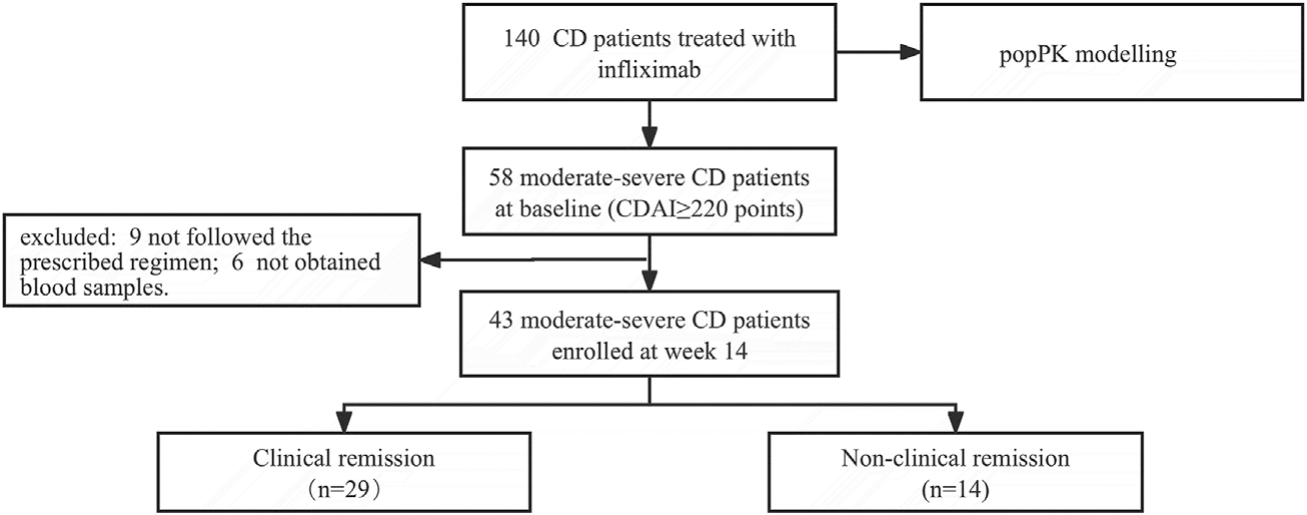
The flow diagram of the cohort study.

In total, 526 blood samples obtained from 140 patients were enrolled to develop the popPK model. Patient characteristics and clinical laboratory results are summarized in Table 1. Of these, 58 patients with CDAI ≥220 points at baseline were followed for evaluating the relationship between early IFX levels and ATI and CR. Fifteen patients were excluded because of inappropriate infusion intervals or a lack of samples (e.g., refused or failed phlebotomy). Finally, 43 consecutive patients with CD were enrolled. The screening and enrolment of the study populations were presented in Figure 1. Furthermore, 29/43 (67.44%) and 14/43 (32.56%) patients in the CR and non-CR groups at week 14 were included, respectively. The baseline characteristics of 43 patients are presented in Table 2. No differences were observed in the baseline characteristics of the patients between both groups.

**TABLE 1:** Summary of demographic and clinical characteristics (n = 140).

Characteristics	Number/Mean ± SD	Min-Max/%
Number of samples	526	
Duration of IFX therapy (weeks), median (IQR)	38.6 (14–105.4)	2–258
Samples below the limit of quantification	66	12.55
ATI		
absence of ATI	292	55.51
1:20	96	18.25
1:60	86	16.35
≥1:180	52	9.89
Gender (Male/Female)	106/34	—
Age^a^ (year, IQR)	34 (24–44)	15–58
Weight^a^ (kg)	59.9 ± 10.8	39–80
Height (cm)	158.5 ± 4.95	150–188
Dose^a^ (mg)		
200/300/400	19/105/16	13.57/75.00/11.43
Albumin^a^ (g/L)	38.3 (34.4–43.1)	27.5–53.1
ESR^a^ (mm/H)	24.5 (12.7–42.7)	1–94

^a^ Before the first infusion of infliximab.

IQR, interquartile range.

**TABLE 2:** Demographic characteristics of the patients according to CDAI at week 14.

Characteristic	CR (n = 29)	Non-CR (n = 14)	*p*
Male, n (%)	23 (79.31)	7 (50.00)	0.08^a^
CDAI points, median (IQR)	241.3 (225.2–291.8)	265.3 (241.5–318.7)	0.16^c^
BMI, kg/m^2^ (median, IQR)	19.03 (17.58–20.82)	18.44 (17.57–19.34)	0.28^d^
Faecal calprotectin>250 μg/g, n(%)	22/26 (84.62)	12/13 (93.31)	>0.99^a^
CRP≥5 mg/L, n(%)	21/25 (84.0)	10/11 (90.91)	>0.99^a^
Albumin, g/L (median, IQR)	38.4 (36.8–41.7)	35.9 (32.1–38.0)	0.12^d^
Non-smoker, n (%)	25 (86.21)	13 (92.86)	>0.99^a^
Previous surgery, n (%)	1 (3.45)	0 (0.0)	>0.99^a^
Date of confirmed CD: A1/A2/A3	0/25/4	2/11/1	0.13^a^
Location: L1/L2/L3/L4a	13/2/13/1	3/4/7	0.16^a^
Behaviour: B1/B2/B3	19/8/2	8/5/1	0.89^a^
Perianal Crohn, n (%)	11 (37.93)	5 (35.71)	0.89^b^
Fistulas, n (%)	10 (34.48)	8 (57.14)	0.16^b^
Previous steroid therapy	2 (6.90)	3 (21.43)	0.31^a^
Previous immunomodulators	5 (17.24)	3 (21.43)	>0.99^a^

^a^ Fisher test.

^b^ Chi-square test.

^c^ Mann–Whitney U test.

^d^ T-test; CDAI, Crohn’s Disease Activity Index; CR, clinical remission; and IQR, interquartile range.

A1, ≤16 years; A2, 17–40 years; A3, >40 years; L1, terminal ileum; L2, colon; L3, ileocolon; L4a, upper gastrointestinal; B1, non-stricturing, non-penetrating; B2, stricturing; B3, penetrating.

### Early induction IFX level and the association of CR

**FIGURE 2:**
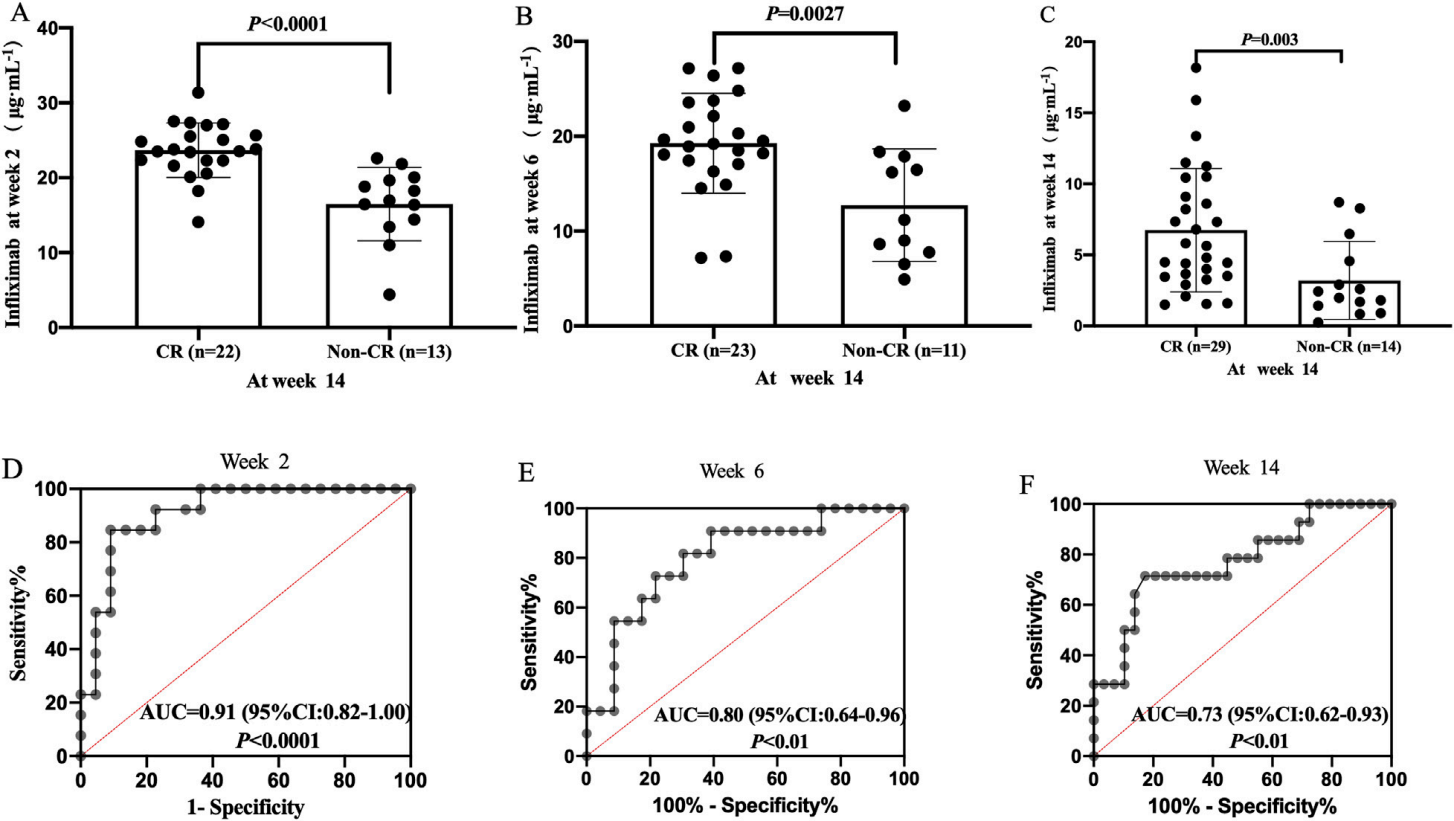
Difference in TLI between the CR and non-CR groups at week 2 **(A)**, week 6 **(B)** and week 14 **(C)**. Correlation between TLI and CR at week 2 **(D)**, week 6 **(E)**; Week 14 **(F)**. CR, clinical remission; TLI, trough levels of infliximab.

The median TLI at week 2, week 6, and week 14 in patients with CR were significantly higher than those in patients without CR [week 2: 23.65 μg/mL (IQR: 22.11–25.99) vs. 16.96 μg/mL (IQR: 13.93–19.86), *P* < 0.0001, Figure 2A; week 6: 19.21 μg/mL (IQR: 17.08–23.57) vs. 11.18 μg/mL (IQR: 7.77–17.88), *P* = 0.0027, Figure 2B; week 14: 5.63 μg/mL (IQR: 3.49–9.77) vs. 2.21 μg/mL (IQR: 1.29–5.05), *P* = 0.003, Figure 2C]. IFX levels >20.08 μg/mL at week 2 (Figure 2D), >18.44 μg/mL at week 6 (Figure 2E), and >3.08 μg/mL at weeks 14 (Figure 2F) were associated with CR. Of these, IFX levels >20.08 μg/mL at week 2 was the key predictor of CR (sensitivity 84.62%, specificity 90.91%, PPV 90.48%, NPV 78.57%; Supplementary Table S1).

### Association between ATI status and IFX levels and CR

**FIGURE 3:**
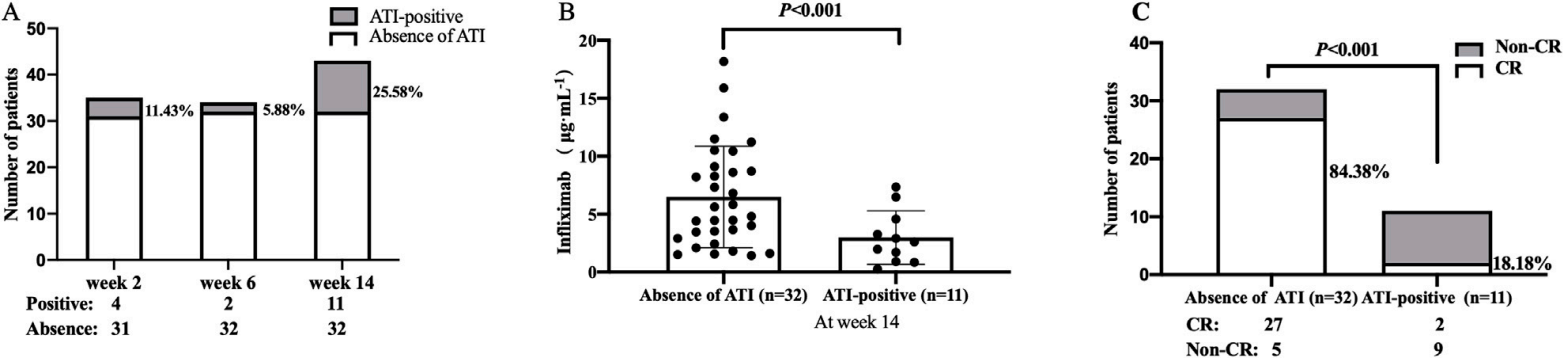
**(A)** ATI incidences at weeks 2, 6, and 14. **(B)** Difference in TLI between the group with and without ATI. **(C)** Association between ATI status and the rate of CR at week 14. CR, clinical remission; TLI, trough levels of infliximab; ATI, antibodies to infliximab.

At weeks 2, 6, and 14, 4 of 35 (11.43%), 2 of 34 (5.88%), and 11 of 43 (25.58%) patients developed ATI, respectively (Figure 3A). The median TLI at week 14 in patients with absence of ATI was significantly higher than those in ATI-positive patients [5.22 μg/mL (IQR: 3.32–8.80) vs. 2.6 μg/mL (IQR: 1.30–3.92), *P <* 0.001, Figure 3B]. Similarly, the rate of CR in patients with absence of ATI was higher than that in ATI-positive patients (84.38% vs. 18.18%, *P <* 0.001; Figure 3C).

### Risk factors of CR

An analysis was conducted to identify potential risk factors of CR at week 14. Univariate analysis revealed significant differences in albumin at baseline, TLI at weeks 2, 6, and 14, and absence of ATI at weeks 2 and 14 between the CR and non-CR groups (*P* < 0.1; Supplementary Table S2). Sex and absence of ATI at weeks 2 and 6 were not associated with CR at week 14 (*P* > 0.1; Supplementary Table S2). In the multivariate analysis, only TLI at week 2 and absence of ATI at week 14 remained statistically significant (*P* < 0.05; Supplementary Table S2).

## Modelling and simulating

### Data source

In total, 526 blood samples obtained from 140 patients were enrolled to develop the popPK model. There were 66 (12.55%) samples below the limit of quantification (BLOQ), and a total of 96.87% of these samples below BLOQ were deemed ATI-positive. The concentration versus time curves of IFX in patients with CD are presented in Supplementary Figure S1.

### Modelling

A one-compartment model with first-order elimination kinetics best described the concentration-time course of IFX. In the covariate screening process, sex, age, height, and ESR had no effects on the PK parameters of IFX. Forward and backward selection revealed that serum albumin concentration and ATI titre had significant effects (Supplementary Tables S3, S4). Therefore, these two covariates were included in the model, and then the final model was subsequently established. Patients with a higher FFM had a lower Vd. IFX Clearance (CL) decreased with lower FFM, higher albumin, and absence of ATI. Vd and CL were 9.65 L and 0.45 L/day, respectively. IFX exhibited inter-individual variability for a CL of 25.1%. PK parameters with significant covariate dependencies in the final model were modelled as follows:

$$V=9.65\times \left(\frac{\text{FFM}}{56.1}\right)$$ (3)

$$\text{CL}=\theta \times 0.45\times {\left(\frac{\text{FFM}}{56.1}\right)}^{0.75}\times {\left(\frac{\text{ALB}}{40}\right)}^{-0.48}\;;\text{ATI}\leq 1:20,\theta =1;\text{ATI}=1:60,\theta =1.17;\text{ATI}\geq 1:180,\theta =1.38;$$ (4)

**FIGURE 4:**
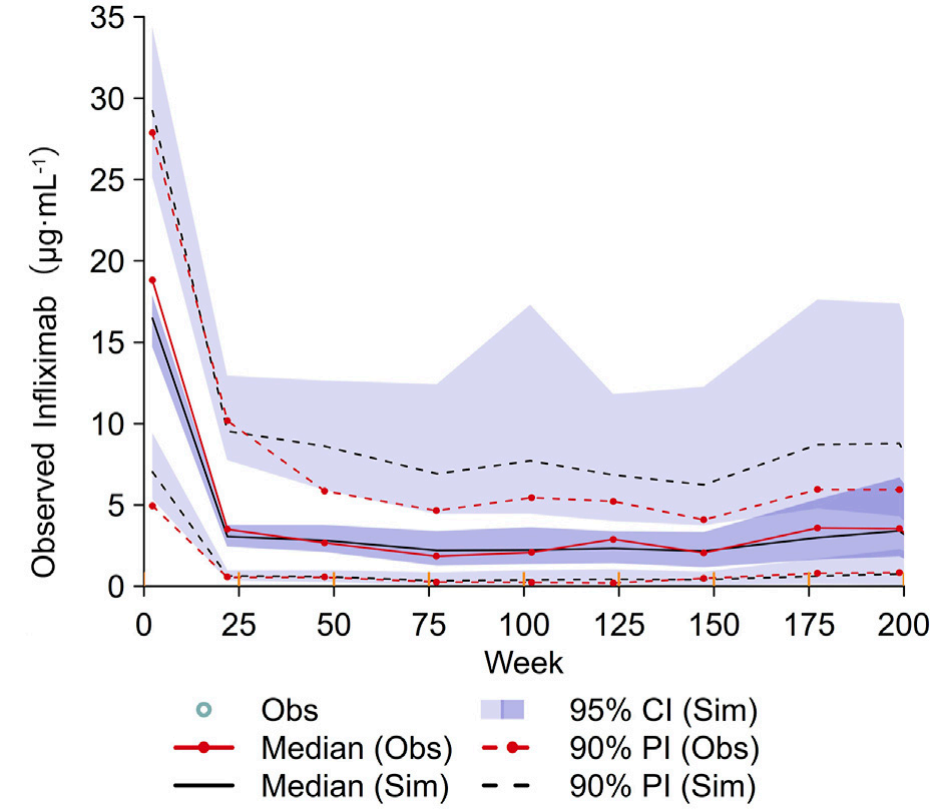
VPC plot of the final model. The red solid lines represent the median observed concentration, and the deep purple fields represent the simulation-based 95% confidence intervals (CIs) for the median. The observed 5th and 95th percentiles are represented by red dashed lines, and the 95% CIs for the corresponding model predicted percentiles are shown as light purple fields. VPC, visual predictive checking.

Parameter estimates from the final and bootstrap models for IFX were summarised in Supplementary Table S5. The point estimates from the original dataset and the median parameter estimates from the bootstrap datasets were similar, indicating the robustness of the final model and the parameter estimates. Goodness-of-fit plots demonstrated that the model adequately described the observed data (Supplementary Figure S2). The VPC demonstrated good agreement between the simulated and observed data (Figure 4). Subgroup VPC plots indicated that the final model from 140 patients was accurate for predicting 43 patients in the prospective cohort study (Supplementary Figure S3).

### Simulating

**FIGURE 5:**
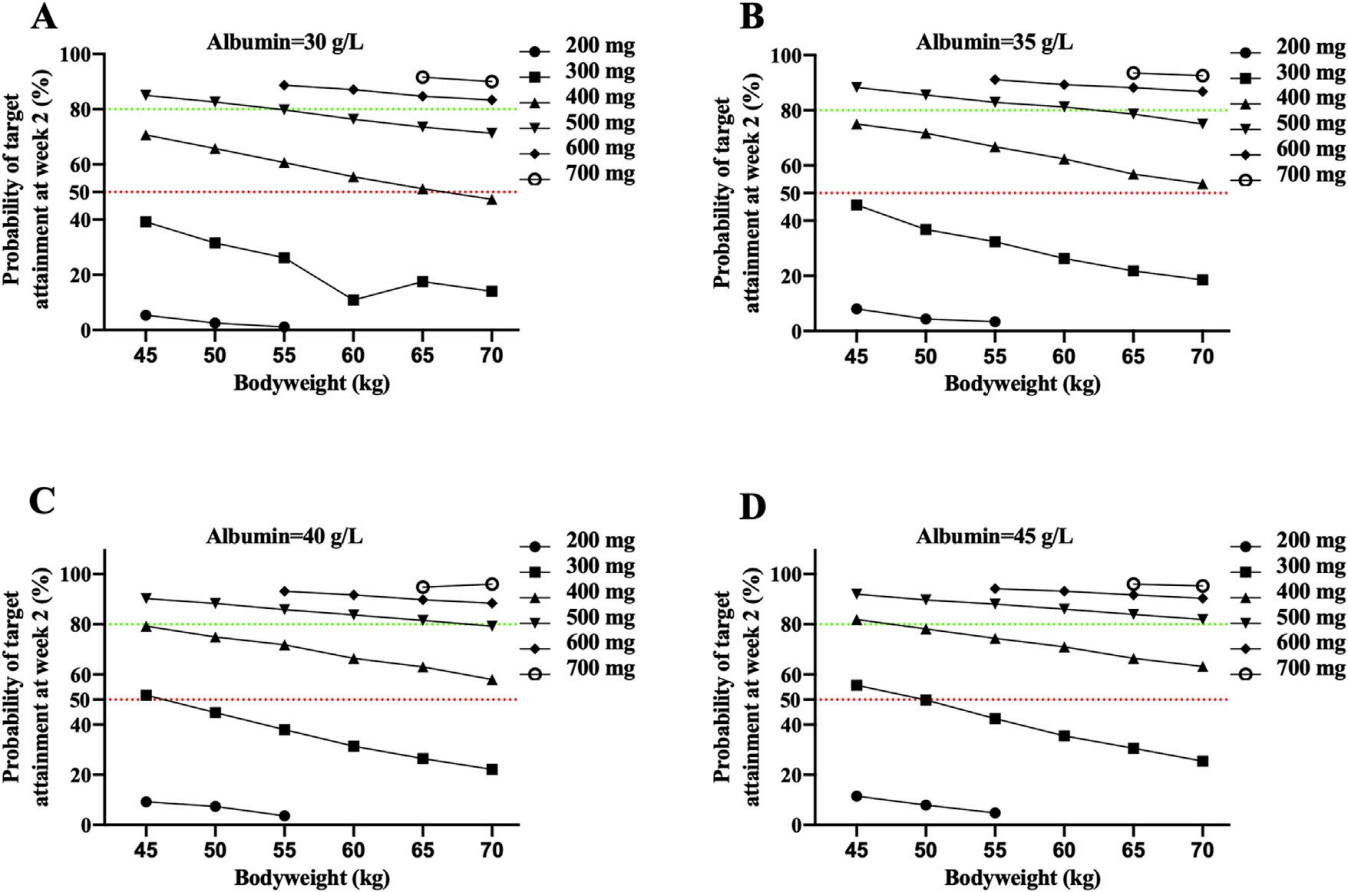
The probability of attaining the identified IFX target concentration of 20.8 μg/mL at week 2 in CD patients with 30 g/L albumin **(A)**, 35 g/L albumin **(B)**, 40 g/L albumin **(C)** and 45 g/L albumin **(D)**. Each dot represents the probability of target attainment based on 1,000 virtual patients with CD in absence of ATI. Red and green horizontal lines reference 50% and 80% target attainment at week 2. Simulations were performed with inter-patient variability.

IFX levels >20.08 μg/mL at week 2 were used as the target. Patients with CD weighing 50–70 kg receiving 300 mg IFX did not achieve >50% probabilities of target attainment (PTA). Only patients weighing 45 kg and a higher albumin concentration (>40 g/L) achieved a higher 50% PTA when treated with 300 mg IFX. Greater than or equal to 400 mg IFX was predicted to be required in over 50% of patients with 45–70 kg and 35–45 g/L albumin, except for patients with 70 kg and 30 g/L albumin (Figure 5).

## Discussion

This study assessed the association of early IFX and ATI with CR. A TLI >20.8 μg/mL at week 2 and absence of ATI at week 14 were associated with CR at the end of the induction period. Our results identified the IFX concentration at week 2 as a key risk factors of CR. With a standard dose of 5 mg/kg, less than 50% of the simulated population with a body weight of 60 kg reached the identified target. A high dose of IFX (over 5 mg/kg) during the first infusion resulted in higher serum IFX concentrations at week 2, especially in patients with low albumin concentrations.

The rate of non-CR in the present study was 32.56%. Similar to our study, Ben-Horin demonstrated that 20%–40% patients with IBD exhibited no clinical improvement at the end of the induction period. The reported incidence of non-CR to IFX varies due to the different definitions and time points (Papamichael et al., 2015). In this study, the CDAI points at week 14 were used as markers of CR, a classic tool to judge efficacy (Thia et al., 2008) instead of the Harvey–Brashow index. Another challenge in RCTs and cohort studies is the lack of active inflammation at baseline (Papamichael et al., 2015). To overcome this problem, patients with moderate-to-severe CD were enrolled in this study. Lastly, IBD type was associated with the rate of CR owing to the heterogeneity of the study populations. Early IFX concentrations for predicting short-term clinical outcomes differ between patients with UC and CD (Gonczi et al., 2017). In this study, patients with CD were enrolled instead of those with UC.

Several studies have linked TLI in induction therapy with clinical outcomes. However, these studies left some questions unanswered. Despite the finding by Dreesen that the cumulative area under the IFX concentration-time curve until endoscopy predicts mucosal healing, this method is time-consuming and ineffective for routine clinical use (Dreesen et al., 2019). Casteele et al. demonstrated that an IFX threshold concentrations at week 2 was associated with endoscopic outcomes at week 8 in patients with UC (Vande Casteele et al., 2019); however, the specificity and the area under the ROC curve were only 25% and 0.57, respectively. Beltrán et al. demonstrated that IFX levels at week 14 could predict primary non-response after IFX therapy induction in patients with CD (Beltrán et al., 2019). Therefore, the timing at week 14 may not be suitable. Peak IFX levels at week 2 and intermediate levels at week 3 correlated with remission at week 30 (Liefferinckx et al., 2022). The IFX levels at these time points requires additional visits. In addition to TLI, changes in IFX clearance during induction therapy might predict treatment outcomes, but it needed an equation of population PK model to estimate clearance (Kantasiripitak et al., 2021). Our study revealed that TLI at week 2 was associated with CR. One advantage of measuring the TLI at week 2 is its simplicity, which allows for easy generalisation.

In addition to the association between early TLI and medium-term responses, several studies on long-term responses have been published. In patients with perianal CD, IFX levels >9.2 μg/mL at week 2 were linked to the closure of fistulas at week 14 and 30 (Davidov et al., 2016). In a paediatric study (CD and UC), TLI >9.2 μg/mL at week 2 predicted CR at week 14, and TLI >2.2 μg/mL at week 6 predicted IFX retention beyond 1 year of treatment (Ungar et al., 2018). Liefferinckx et al. demonstrated that early IFX concentration was associated with long-term response (Liefferinckx et al., 2017). Park demonstrated that an IFX concentration >4.5 μg/mL at week 6 predict CR at week 30 in active patients with CD (Park et al., 2023). Conversely, no direct relationship was observed between TLI at week 6 and the persistence of IBD-related peripheral arthralgia (Levartovsky et al., 2020). Many factors may influence the long-term response to IFX; therefore, early IFX concentration is more closely related to medium-term response.

Few studies have focused on immunogenicity during the induction period in patients with IBD. The detection of ATI during the induction period is challenging due to the high concentration of IFX in the plasma. Tournier et al. (2021) demonstrated that ATI using a drug-tolerant assay at week 2 was detected in 67% of patients and that the prevalence of ATI at week 2 predicted further IFX discontinuation (Kantasiripitak et al., 2023). If immunoassays for ATI have limitation in terms of drug-tolerance, monitoring the clearance of IFX could help in identifying the presence of ATI that are not detectable by these assays (Kantasiripitak et al., 2021). In this study, ATI was detected at week 2 in only 11.43% of the patients because of the limited sample size and assay method. Absence of ATI at week 14 has been demonstrated to be an independent risk factor for CR.

A recent real-world study highlighted the advantages of using a model-based tool as a highly promising approach for informing dosing choices. Specifically, it demonstrated that the tool in minimising ATI formation and ensuring the continued efficacy of IFX therapy during induction is valuable (Dubinsky et al., 2022). Moreover, model-based tools using the IFX concentration at week 6 improved deep remission rates in children with IBD during induction (Kantasiripitak et al., 2023). Although in the PK modeling, the dose range was 200–400 mg, the dose range of 200 to 700 mg of IFX was selected for the simulation study based on the linear PK characteristics described in the FDA drug label. A higher IFX dose was associated with an increased probability of attaining the identified exposure target, particularly in patients with low albumin levels. An accelerated IFX induction regimen was also beneficial for patients with acute severe UC (Gibson et al., 2015). Additional infusion visits may affect the patient’s quality of life. A high dose of IFX (>10 mg/kg) may be associated with more serious infusion events (Hendler et al., 2015).

Our study had a few limitations. First, our cohort was restricted to only one tertiary centre, and multi-centre studies are required to validate our findings in the future. Second, we did not assess endoscopic data or markers of mucosal healing, such as faecal calprotectin levels. Third, the albumin levels increased slightly due to disease improvement. The PTA increased with increasing albumin levels. Lastly, the inflammatory burden played a more significant role in CR than IFX exposure (Billiet et al., 2017), we did not detect cytokines and inflammatory biomarkers.

In conclusion, this study demonstrated that a higher TLI at week 2 and absence of ATI at week 14 were associated with CR. TLI >20.8 μg/mL at week 2 could serve as a prognostic factor for predicting the success of induction, whereas lower levels could prompt the physician for additional interventions.

## Data Availability

The original contributions presented in the study are included in the article/Supplementary Material, further inquiries can be directed to the corresponding authors.
