# Supplementary material for: Optimising infliximab induction dosing to achieve clinical remission in Chinese patients with Crohn’s disease
Source: Front Pharmacol. 2024 Aug 27;15:1430120. doi: 10.3389/fphar.2024.1430120 (PMC11384982; doi:10.3389/fphar.2024.1430120)
Supplement: Supplementary file 1 [file DataSheet1.docx]

**Optimising Infliximab Induction Dosing to Achieve Clinical Remission in Chinese Patients with Crohn’s Disease**

**Kouzhu Zhu^1,2†^, Xiaoliang Ding^1,4†^, Ling Xue^1,4†^,Linsheng Liu^1,4^,Yan Wang^2^,Yun Li^1^, Qinhua Xi^3^, Xueqin Pang^3^, Weichang Chen^3*^, Liyan Miao^1,5*^**

**†These authors contributed equally to this work and share first authorship.**

**1.Department of Pharmacy, The First Affiliated Hospital of Soochow University, Suzhou, China, 2.Department of Pharmacy, Affiliated Children’s Hospital to Jiangnan University(Wuxi Children's Hospital), Wuxi, China, 3.Department of Gastroenterology, The First Affiliated Hospital of Soochow University, Suzhou, China, 4.Institute for Interdisciplinary Drug Research and Translational Sciences, Soochow University, Suzhou, China, 5.National Clinical Research Center for Hematologic Diseases, The First Affiliated Hospital of Soochow University, Suzhou, China**

**Corresponding author information**

**Liyan Miao:miaolysuzhou@163.com**

**Weichang Chen:weichangchen@126.com**

**Table S1** Accuracy of IFX levels for predicting CR at week 14

| Week | Cut-off value (μg/mL) | Sensitivity  (%) | Specificity  (%) | PPV  (%) | NPV  (%) |
| --- | --- | --- | --- | --- | --- |
| 2 | 20.08 | 84.62 | 90.91 | 90.48 | 78.57 |
| 6 | 18.44 | 90.91 | 60.87 | 93.33 | 52.63 |

Note: CR, clinical remission; PPV: positive predictive value; NPV: negative predictive value.

**Table S2** Factors associated with CR

| Variables | Univariate analysis | | Multivariate analysis | |
| --- | --- | --- | --- | --- |
|  | OR (95% CI) | *P* | OR (95% CI) | *P* |
| Sex (male) | 3.83(0.96-15.24) | 0.06 |  |  |
| Albumin at baseline | 1.23 (1.03-1.48) | 0.02 |  |  |
| TLI at week 2 | 1.63 (1.18-2.25) | 0.003 | 1.55 (1.07-2.25) | 0.02 |
| Absence of ATI at week 2 | 7.36 (0.69-78.71) | 0.099 |  |  |
| TLI at week 6 | 1.23 (1.05-1.43) | 0.009 |  |  |
| Absence of ATI at week 6 | 2.20 (0.13-38.83) | 0.59 |  |  |
| TLI at week 14 | 1.38 (1.06-1.81) | 0.018 |  |  |
| Absence of ATI at week 14 | 24.3(3.99-174.47) | 0.001 | 18.23(1.02-338.32) | 0.04 |

Note: CR, clinical remission; OR, odd ratio;CI, confidence interval; TLI, trough levels of infliximab; ATI, antibodies to infliximab.


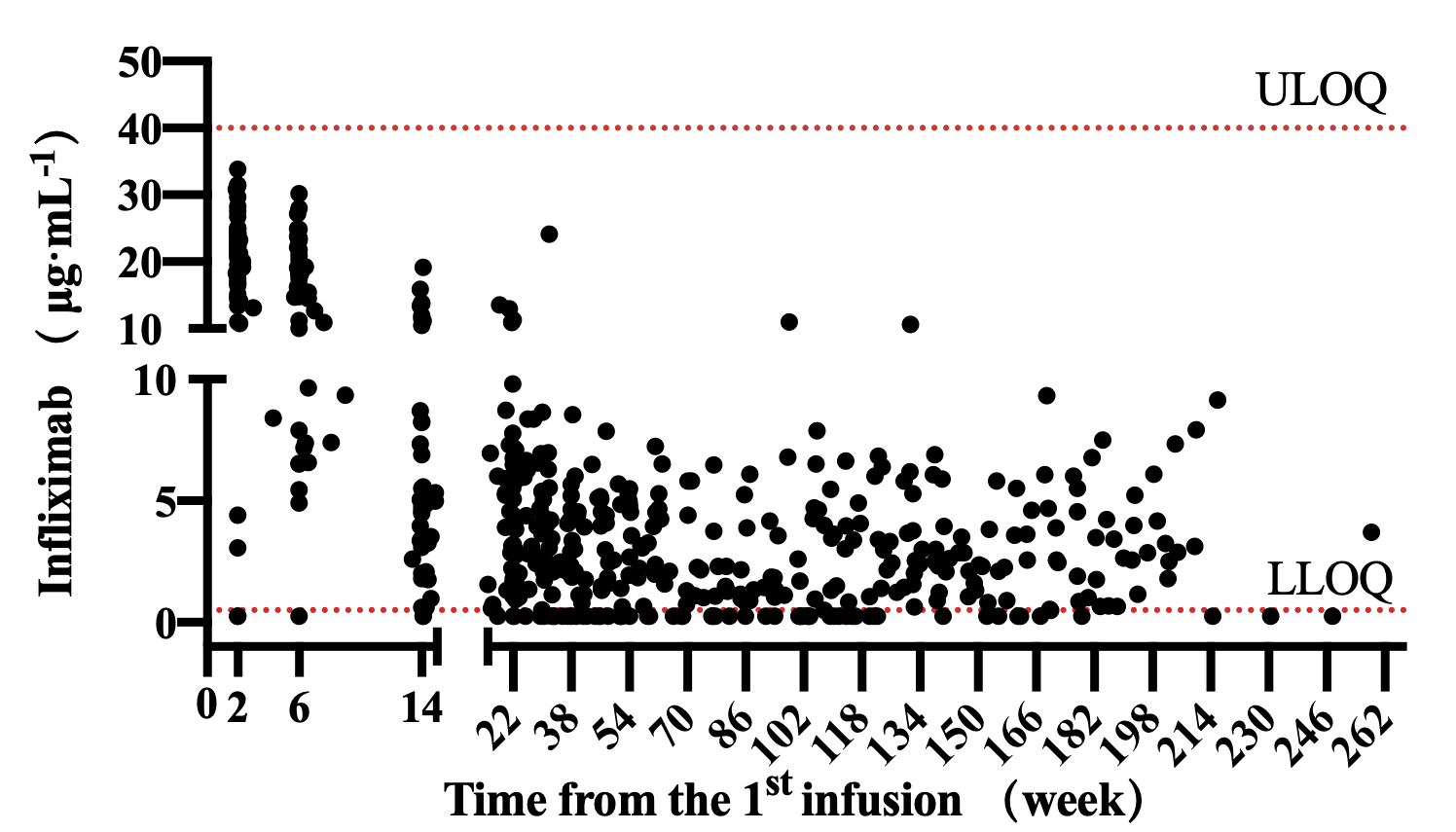


Figure S1. Concentration vs. time curves of IFX in Crohn’s disease. LLOQ, lower limit of quantitation; ULOQ, upper limit of quantitation.

Table S3 The result of Bootstrap for estimating confidence interval of parameters

| Covariates | Median | 2.5th percentile | 97.5th percentile | Status in the model |
| --- | --- | --- | --- | --- |
| Age_CL | -0.02 | -0.21 | 0.19 | Remove |
| Sex_CL | 1.18 | 0.95 | 1.51 | Remove |
| Albumin_CL | -0.43 | -0.94 | -0.04 | Keep |
| ESR_CL | 0.05 | -0.01 | 0.10 | Remove |
| ATI (≤1:20)_CL | 1.05 | 0.99 | 1.12 | Remove |
| ATI (1:60)_CL | 1.17 | 1.06 | 1.28 | Keep |
| ATI (≥1:180)_CL | 1.37 | 1.15 | 1.66 | Keep |
| Age_Vd | -0.19 | -0.45 | 0.10 | Remove |
| Sex_Vd | 1.26 | 0.98 | 1.51 | Remove |
| Albumin_Vd | -0.19 | -1 | 0.54 | Remove |
| ESR | -0.02 | -0.11 | 0.05 | Remove |

Note: CL, Clearance; ATI, antibodies to infliximab; ESR, erythrocyte sedimentation rate; Vd, distribution volume.

Table S4 The results of backward elimination method for covariate selection

| Model | Model Description | OFV | ΔOFV | df | *P* |
| --- | --- | --- | --- | --- | --- |
| 1 | Final model | 943.35 |  |  |  |
| 2 | Model 1-FFM on V | 992.19 | 48.83 | 1 | <0.001 |
| 3 | Model 1-FFM on CL | 956.79 | 13.43 | 1 | <0.001 |
| 4 | Model 1-Albumin on CL | 964.78 | 21.42 | 1 | <0.001 |
| 5 | Model 1-ATI (1:60) on CL | 978.13 | 34.78 | 1 | <0.001 |
| 6 | Model 1-ATI (1:180) on CL | 1 003.79 | 60.44 | 1 | <0.001 |

Note: CL, Clearance ; ATI, antibodies to infliximab; OFV, objective function value.

Table S5 Parameter estimates from the final model and Bootstrap results

| Parameter | Estimate | RSE (%) | Bootstrap | | | |
| --- | --- | --- | --- | --- | --- | --- |
|  |  |  | Median estimate | 2.5th percentile | 97.5th percentile | RSE (%) |
| CL (L/d) | 0.45 | 3.8 | 0.45 | 0.42 | 0.48 | 3.6 |
| Albumin on CL | -0.48 | -27.9 | -0.49 | -0.74 | -0.25 | -27.4 |
| ATI (1:60) on CL | 1.17 | 4.2 | 1.16 | 1.04 | 1.27 | 4.8 |
| ATI (≥1:180) on CL | 1.38 | 7.5 | 1.37 | 1.16 | 1.69 | 14.8 |
| V (L) | 9.65 | 4.2 | 9.59 | 8.87 | 10.5 | 4.2 |
| IIV_CL(%) | 25.1 | 4.3 | 25.2 | 20.9 | 30.5 | 9.6 |
| IIV_CL shrinkage (%) | 5.9 |  |  |  |  |  |
| Residual variability |  |  |  |  |  |  |
| Proportional error (%) | 26.6 | 11.2 | 25.9 | 17.9 | 32.1 | 13.6 |
| Additive error, μg/mL | 0.15 | 38 | 0.16 | 0.08 | 0.36 | 52.9 |
| ε1 shrinkage (%) | 10.9 |  |  |  |  |  |

Note: CL, clearance; Vd, volume of distribution; IIV: inter-individual variability;RSE, relative standard error.


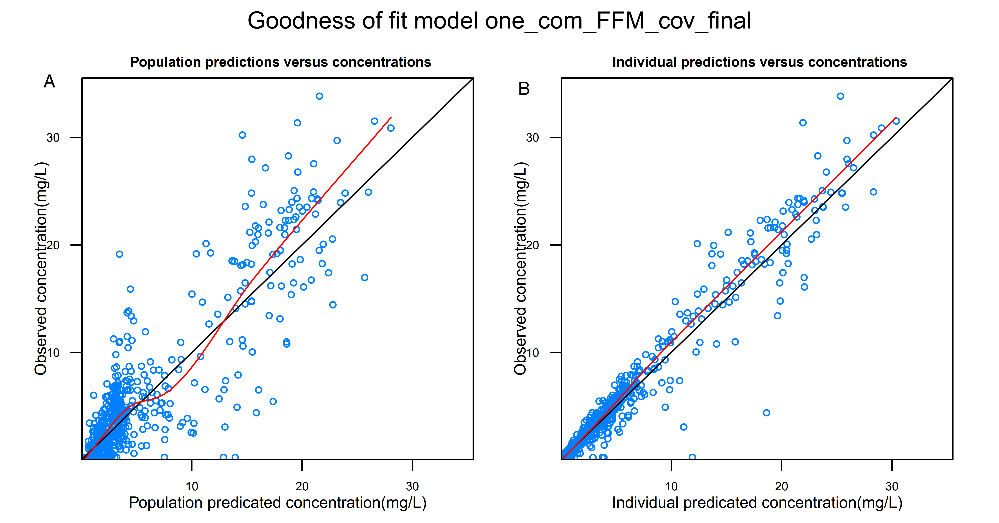


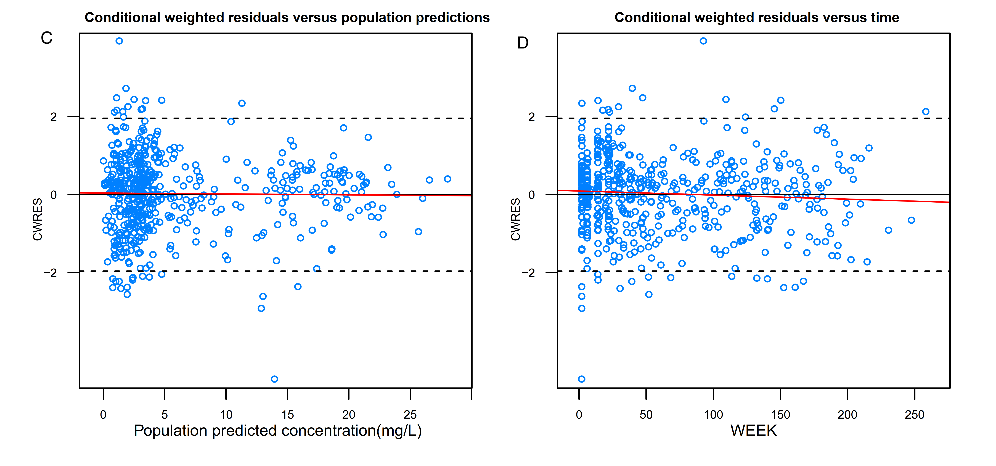
Figure S2. Goodness-of-fit plots for the final model. A. Observed concentration (DV) Versus population predicted concentration (PRED); B. DV versus individual predicted concentration (IPRED); C. Conditional weighted residuals (CWRES) versus PRED; D. CWRES Versus time after the last dose. The red solid lines in A and B are identity lines, and the red solid lines in C and D are zero lines.


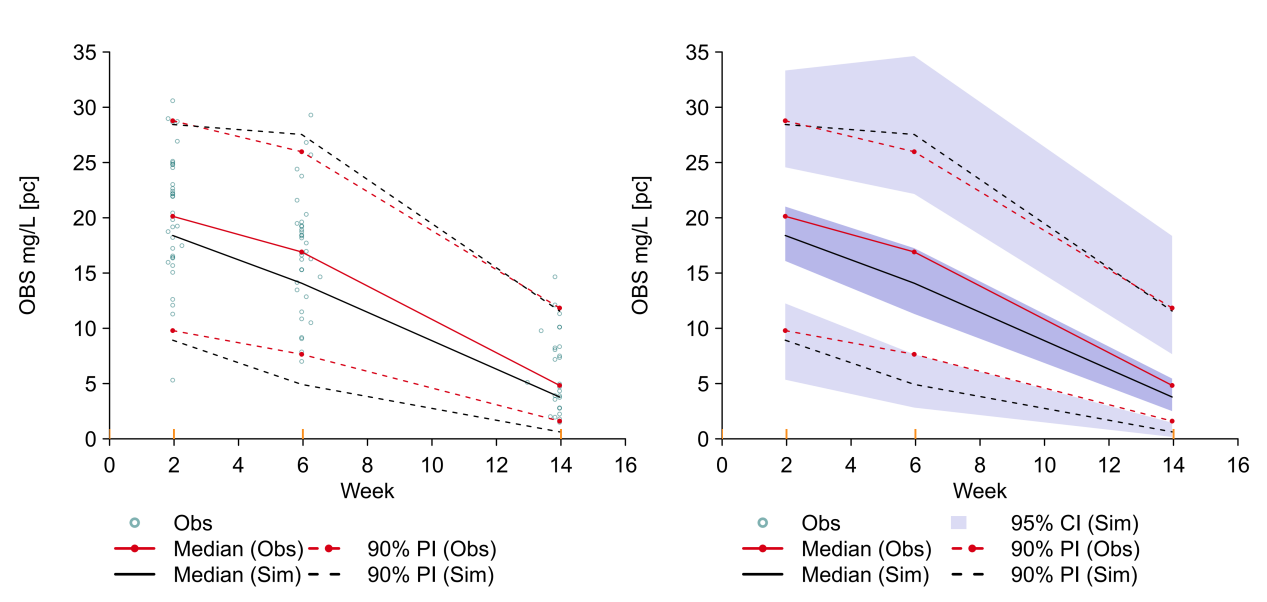


Figure S3. Subgroup VPC plot of the final model in the 43 subjects from the prospective cohort study. The red solid lines represent the median observed concentration, and the deep purple fields represent the simulation-based 95% confidence intervals (CIs) for the median. The observed 5th and 95th percentiles are represented by red dashed lines, and the 95% CIs for the corresponding model predicted percentiles are shown as light purple fields.VPC,visual predictive checking.
